# A Markov blanket-based method for detecting causal SNPs in GWAS

**DOI:** 10.1186/1471-2105-11-S3-S5

**Published:** 2010-04-29

**Authors:** Bing Han, Meeyoung Park, Xue-wen Chen

**Affiliations:** 1 Bioinformatics and Computational Life Sciences Laboratory, ITTC, Department of Electrical Engineering and Computer Science, The University of Kansas, 1520 West 15th Street, Lawrence, KS 66045, USA

## Abstract

**Background:**

Detecting epistatic interactions associated with complex and common diseases can help to improve prevention, diagnosis and treatment of these diseases. With the development of genome-wide association studies (GWAS), designing powerful and robust computational method for identifying epistatic interactions associated with common diseases becomes a great challenge to bioinformatics society, because the study of epistatic interactions often deals with the large size of the genotyped data and the huge amount of combinations of all the possible genetic factors. Most existing computational detection methods are based on the classification capacity of SNP sets, which may fail to identify SNP sets that are strongly associated with the diseases and introduce a lot of false positives. In addition, most methods are not suitable for genome-wide scale studies due to their computational complexity.

**Results:**

We propose a new Markov Blanket-based method, DASSO-MB (Detection of ASSOciations using Markov Blanket) to detect epistatic interactions in case-control GWAS. Markov blanket of a target variable T can completely shield T from all other variables. Thus, we can guarantee that the SNP set detected by DASSO-MB has a strong association with diseases and contains fewest false positives. Furthermore, DASSO-MB uses a heuristic search strategy by calculating the association between variables to avoid the time-consuming training process as in other machine-learning methods. We apply our algorithm to simulated datasets and a real case-control dataset. We compare DASSO-MB to other commonly-used methods and show that our method significantly outperforms other methods and is capable of finding SNPs strongly associated with diseases.

**Conclusions:**

Our study shows that DASSO-MB can identify a minimal set of causal SNPs associated with diseases, which contains less false positives compared to other existing methods. Given the huge size of genomic dataset produced by GWAS, this is critical in saving the potential costs of biological experiments and being an efficient guideline for pathogenesis research.

## Background

Compared to Mendelian disorders that are monogenic and rare in population, some common complex diseases like various types of cancers, diabetes and hypertension are conjectured to be caused by two types of interactions related with multiple genetic factors: gene-gene interactions and gene-environment interactions [1]. Interactions between genes or single nucleotide polymorphisms (SNPs) in chromosomal regions are called * epistasis *[2-4]. Detecting epistasis associated with complex and common diseases became an important issue in human genetics and can build a new pavement towards the improvement of prevention, diagnosis and treatment of these diseases.

Recent development of high-throughput technologies has made it possible to produce a huge amount of genotype data and contribute to the analysis of genome-wide association studies (GWAS) [5-7]. Furthermore, the international HapMap project has been used to support GWAS actively by the analysis of the common patterns of DNA sequence variations in different populations [8,9]. However, the number of SNPs from case-control GWAS is typical more than 10 million and using traditional epistatic interactions detection methods such as parametric regression to identify multiple loci causing diseases simultaneously among all possible combinations of SNPs is inappropriate from genome-wide case-control data. Therefore, designing robust and manageable methods to address this mathematical and computational problem presents a great challenge to scientists in bioinformatics.

By far, a number of statistical methods have been proposed to detect epistatic interactions. Among these, the most commonly-used parametric statistical method is logistic regression [10]. Logistic regression models the probability of a disease as a linear function of independent SNPs (SNPs are expressed as ternary variables) and finds an optimal logical SNP set associated with the disease status by simulated annealing algorithm [11]. When used for modelling high-order interactions, logistic regression methods relates to many empty contingency-table cells, which often leads to very large standard errors for parameter estimation and therefore increases the type I errors. Meanwhile, if the number of samples is small, high-order interaction models creates a large number of parameters and often results in an overfitting problem. To overcome these problems in logistic regression, Richie *et al* proposed and developed a multifactor dimensionality reduction (MDR) method [12-15]. MDR first constructs a contingency table for every possible set of SNPs and then labels the cells of the table “high risk” or “low risk” based on the cases/control ratio of each cell. By the label of each cell in the contingency table, MDR runs 10-fold cross-validation to select an SNP set with the smallest prediction error and/or the largest consistency. The merit of MDR compared to other statistical methods is that MDR is non-parametric and model-free. However, it has two fundamental limitations: MDR selects the *k*-way interactions purely by the prediction performance and moreover it employs an exhaustive searching strategy to avoid local optima, which makes it impractical for large-scale datasets. Therefore, when applied to large-scale datasets, MDR requires to use some feature selection methods such as ReliefF [16] as a filter for the top N SNPs, which will affect the performance of MDR significantly. Park and Hastie [17] made efforts to detect gene-gene interactions using a forward stepwise method based on penalized logistic regression (stepPLR). However, regression methods are typically computationally expensive because of the time needed for parameter estimations. Although stepPLR adopted forward selection and penalization to choose the causal SNPs, it can not overcome the essential limitations of regression. Recently, Zhang and Liu proposed a Bayesian epistasis association mapping (BEAM) method [18]. BEAM is a Bayesian marker partition model using Markov Chain Monte Carlo to reach an optimal marker partition with the highest posterior probability and a new B statistic instead of the conventional *x*^2^ statistic to check each marker or set of markers for significant associations with the disease. Despite their success to some degrees, statistical methods can only be applied to small-scale analysis due to their computational complexity.

The alternative approaches for statistical methods are machine-learning methods since detecting epistatic interactions is highly related to feature selection problem. Chen *et al*. proposed a support vector machine approach for detecting gene-gene interactions based on RFE (recursive feature elimination), RFA (recursive feature addition) and GA (genetic algorithm) feature selection methods [19]. Jiang *et al*. adopted random forests, which is an ensemble learning technique, to the detection of epistatic interactions in case-control studies [20]. They first ranked SNPs based on gini importance of each SNP from random forests and then performed a greedy search for a small subset of SNPs that could minimize the classification error by a Sliding Window Sequential Forward feature Selection (SWSFS) algorithm. The common limitation of machine learning-based methods is that they typically identify a SNP set that produces the highest classification accuracy, but not necessarily has the strongest association with the diseases. As a result, machine learning-based approaches tend to introduce many false positives, since the including of more SNPs increases classification accuracies.

In this paper, we propose a new Markov Blanket-based method, DASSO-MB (Detection of ASSOciations using Markov Blanket) to detect epistatic interactions in case-control studies. The Markov Blanket is a minimal set of variables, which can completely shield the target variable from all other variables based on Markov condition property. Thus we can guarantee that the SNP set detected by DASSO-MB has a strong association with diseases and contains fewest false positives. Furthermore, DASSO-MB performs a heuristic search by calculating the association between variables to avoid the time-consuming training process as in SVMs and Random Forests.

We compare DASSO-MB with four other commonly used methods (BEAM [18], SVM [19], MDR [12-15] and stepPLR [17]) on simulated datasets generated from three disease models [10,18,20]. The results show that DASSO-MB significantly outperforms other methods and is capable of finding SNPs strongly associated with diseases. For genome-wide case-control datasets, we use the Age-related Macular Degeneration (AMD) dataset containing 116,204 SNPs genotyped for 96 cases and 50 controls [7]. DASSO-MB can find the AMD associated SNP rs380390 in the result SNP set and this demonstrates the power and scalability of DASSO-MB.

## Results and discussion

### Epistatic models and simulation study

We first evaluate the proposed DASSO-MB on simulated data sets, which are generated from three commonly-used disease models developed elsewhere [10,18]. We show the three disease models in Table 1. In each cell of the table are the disease odds for each genotype combination at two loci (A and B), where *α* is the baseline effect and *θ* is the genotypic effect. In model 1, two disease loci contribute to the disease risk independently and produce additive effects. In model 2, the disease risk is presented only when both loci have at least one disease allele. Model 3 is a threshold model and is similar to model 2 except that additional disease alleles at each locus do not further increase the disease risk.

**Table 1:** Three disease models

Model 1	AA	Aa	aa
BB	*α*	*α* (1+ *θ*)	*α* (1+ *θ*)^2^
Bb	*α* (1+ *θ*)	*α* (1+ *θ*)^2^	*α* (1+ *θ*)^3^
bb	*α* (1+ *θ*)^2^	*α* (1+ *θ*)^3^	*α* (1+ *θ*)^4^

**Model 2**	**AA**	**Aa**	**aa**

BB	*α*	*α*	*α*
Bb	*α*	*α* (1+ *θ*)	*α* (1+ *θ*)^2^
bb	*α*	*α* (1+ *θ*)^2^	*α* (1+ *θ*)^4^

**Model 3**	**AA**	**Aa**	**aa**

BB	*α*	*α*	*α*
Bb	*α*	*α* (1+ *θ*)	*α* (1+ *θ*)
bb	*α*	*α* (1+ *θ*)	*α* (1+ *θ*)

To generate data, we need to determine three parameters associated with each model: the marginal effect of each disease locus (*λ*), the minor allele frequencies (MAF) of both disease loci, and the strength of linkage disequilibrium (LD) between the unobserved disease locus and a genotyped locus. LD is a non-random association of alleles at different loci and is quantified by the squared correlation coefficient *r*^2^ calculated from allele frequencies [21]. The prevalence of a disease is the proportion the total number of cases of the disease in the population and in this paper we assume that the disease prevalence is 0.1 for all these three disease models [10]. The marginal effect of each disease locus (*λ*) can be determined by the baseline effect *α* and the genotypic effect *θ* in Table 1 and the minor allele frequencies (MAF) of both disease loci. So first we fix *λ*, the disease prevalence and MAF of both disease loci. Then we numerically derive the model parameters *θ* and *α*. Based on *θ* and *α*, we calculate the conditional probability of each genotype combination given disease status which is necessary for generating data [22]. In this paper, we set parameters for each model as follows:

• Model1: *λ* =0.3; *r*^2^ =0.7,1.0; MAF=0.05, 0.1, 0.2, 0.5.

• Model2: *λ* =0.3; *r*^2^ =0.7,1.0; MAF=0.05, 0.1, 0.2, 0.5.

• Model3: *λ* =0.6; *r*^2^ =0.7,1.0; MAF=0.05, 0.1, 0.2, 0.5.

For each non-disease marker, we randomly chose its MAF from a uniform distribution in [0.0. 0.5]. We generate 50 datasets and each dataset contains 100 markers genotyped for 1,000 cases and 1,000 controls based on each parameter setting for each model.

We compare the DASSO-MB algorithm with four commonly used methods: BEAM, Support Vector Machine, MDR and stepPLR on the three simulated disease models. We use power as our evaluation criterion, which is defined as the proportion of simulated datasets in which only two diseases associated markers are identified without any false positives, to measure the performance of each method.

BEAM uses a Bayesian marker partition model to partition SNPs into three groups: group 0 contains markers unlinked to the disease, group 1 contains markers contributing independently to the disease, and group 2 contains markers that jointly influence the disease. After the partition step by MCMC, candidate SNPs or groups of SNPs are further filtered by the B statistic [18]. The BEAM software is downloaded from http://www.fas.harv-ard.edu/~junliu/BEAM.

For support vector machines, we use LIBSVM with a RBF kernel to detect gene-gene interactions [23]. A grid search is used for selecting optimal parameters. Instead of using the exhaustive greedy search strategy for SNPs as in [19], which is very time-consuming and infeasible to large-scale datasets, we turn to a search strategy used in [20]. First we rank SNPs based on the mutual information between SNPs and disease status label that is 0 for the control and 1 for the case. Then, we use a sliding window sequential forward feature selection (SWSFS) algorithm in [20] based on SNPs rank. The window size in SWSFS algorithm determines how robust the algorithm could be and we set it to 20.

Since MDR algorithm can not be applied to a large dataset directly, we first select top 10 SNPs by ReliefF [16], a commonly-used feature selection algorithm, and then MDR performs an exhaustive search for a model consisting of no more than four SNPs that can maximize cross-validation consistency and prediction accuracy. When one model has the maximal cross-validation consistency and another model has the maximal prediction accuracy, MDR follows statistical parsimony (selects the model with fewer SNPs).

For stepPLR, we download the R package from CRAN (ftp://200.17.202.1/CRAN/ web/packages/stepPlr). StepPLR provides both stepwise forward and backward methods for feature selection procedure. We use both methods and set the regularization parameter *λ* to default value (10^-4^) for the L2 norm of the coefficients.

**Figure 1:**
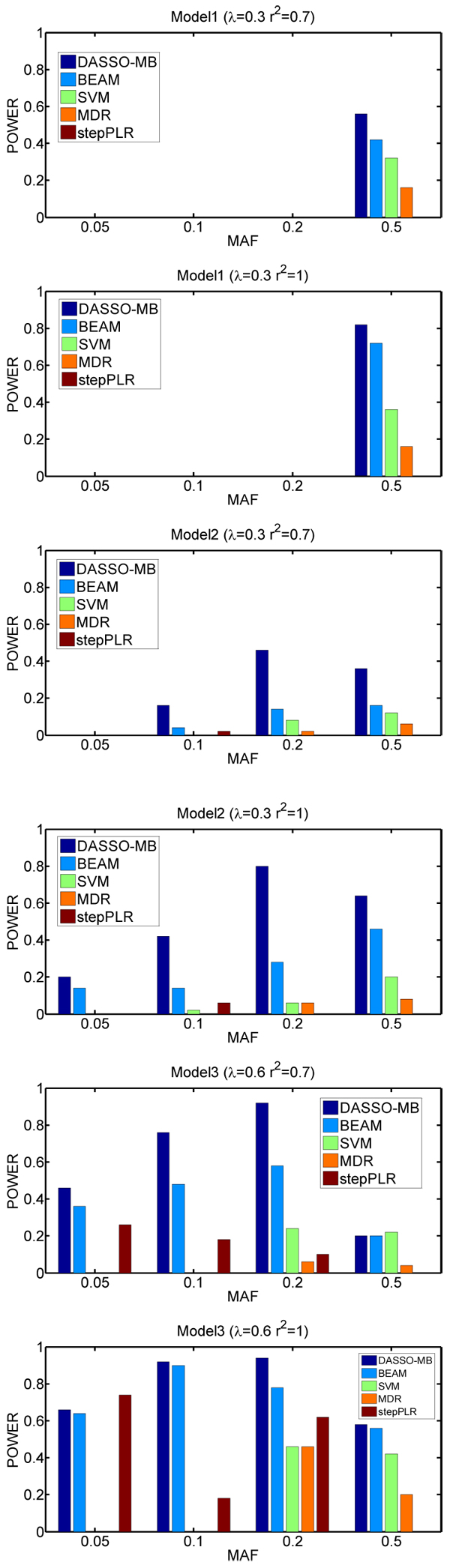
**Performance comparison** The power is defined as the proportion of simulated datasets whose result only contains two disease associated markers without any false positives.

The results on the simulated data are shown in Figure 1. As can be seen, among the five methods, the DASSO-MB algorithm performs the best. BEAM is the second best. Interestingly, BEAM prefers to assign the two disease-associated markers to group 1, which means that BEAM considers that the two disease SNPs affect the disease independently. In most cases, the powers of both MDR and SVM are much smaller than those of the DASSO-MB and BEAM algorithms. For the MDR algorithm, the poor performance may be due to the use of ReliefF to reduce SNPs from a very large dimensionality.

In some other studies, the definition of power is not in a strict sense. For example, in [18,20] the power is defined as the proportion of 50 data sets in which all associated markers are identified at a significance threshold of 0.1 after Bonferroni correction. In other words, false positives are allowed in the final SNP sets. Accordingly, we also evaluate the methods in terms of the power defined as the proportion of simulated datasets in which two diseases associated markers are identified with no more than two false positives. The results of those three models are shown in Table 2. In parentheses we list the average number of false positives. From Table 2, we can see that the DASSO-MB again outperforms other algorithms. Furthermore, the DASSO-MB algorithm finds SNP sets with fewer false positives. Compared to the strict definition of power, a difference we can see is that for MAF > 10%, SVM actually detects the two disease associated markers in more datasets than BEAM, however, at the cost of introducing more false positives.

**Table 2:** Comparison of performance of DASSO_MB, BEAM and SVM algorithms

	MAF		
**Model 1 (*r*^2^=0.7)**	0.05	0.1	0.2	0.5
DASSO-MB	0(0)	0(0)	0(0)	32(0.16)
BEAM	0(0)	0(0)	0(0)	22(0.05)
SVM	1(3)	1(3)	0(0)	33(0.7)

	**MAF**		

**Model 1 (*r*^2^=1)**	0.05	0.1	0.2	0.5
DASSO-MB	0(0)	0(0)	0(0)	46(0.11)
BEAM	0(0)	0(0)	0(0)	36(0.07)
SVM	0(0)	0(0)	1(2)	43(0.76)

	**MAF**		

**Model 2 (*r*^2^=0.7)**	0.05	0.1	0.2	0.5
DASSO-MB	0(0)	8(0)	26(0.12)	18(0)
BEAM	0(0)	2(0)	10(0.3)	9(0.11)
SVM	0(0)	2(1.5)	14(0.93)	21(0.8)

	**MAF**		

**Model 2 (*r*^2^=1)**	0.05	0.1	0.2	0.5
DASSO-MB	10(0)	22(0.05)	42(0.05)	33(0.03)
BEAM	8(0.13)	7(0)	17(0.24)	27(0.11)
SVM	1(2)	3(0.67)	22(1.18)	33(0.94)

	**MAF**		

**Model 3 (*r*^2^=0.7)**	0.05	0.1	0.2	0.5
DASSO-MB	24(0.04)	44(0.14)	47(0.02)	11(0.09)
BEAM	21(0.14)	24(0)	32(0.09)	11(0.09)
SVM	1(1)	6(1.83)	29(0.83)	29(0.83)

	**MAF**		

**Model 3 (*r*^2^=1)**	0.05	0.1	0.2	0.5
DASSO-MB	34(0.03)	50(0.08)	49(0.04)	31(0.06)
BEAM	33(0.03)	47(0.04)	43(0.09)	31(0.1)
SVM	5(1.6)	23(1.52)	42(0.64)	38(0.55)

We show the number of datasets in which two disease-associated markers can be identified with no more than two false positives. The average number of false positives is in the parentheses.

### Application to real data

From the results on simulated data with 100 SNPs, DASSO-MB demonstrates a better performance than four other methods. Notice that a real genome-wide case-control association study may require genotyping of 30,000–1,000,000 common SNPs. In this section, we show that DASSO-MB algorithm can also handle large-scale datasets in real genome-wide case-control studies. We consider an Age-related Macular Degeneration (AMD) dataset, which contains 116,204 SNPs genotyped with 96 cases and 50 controls [7]. AMD (OMIM 603075) [24] is a common genetic disease related to the progressive visual dysfunction in age over 70 in the developed country. A GWA study was successfully conducted on this disease finding two associated SNPs, rs380390 and rs1329428 (‘rs’: assigned reference SNP ID by dbSNP [25]) in non-coding region of the gene for complement factor H (*CFH*), which is located on chromosome 1 in a region linked to AMD [7].

In the phase of preprocessing data, we remove non-polymorphic SNPs and those that significantly deviated from Hardy-Weinberg Equilibrium (HWE). We also remove all SNPs that have more than five missing genotypes. After filtering, there are 91,495 SNPs lying in 22 autosomal chromosomes remained.

DASSO-MB detects two associated SNPs. The first one is SNP rs380390, which is already found in [7] with a significant association with AMD. The other SNP detected by the DASSO-MB algorithm is SNP rs1374431, which is also located in a non-coding region between LOC644301 and KIAA1715 in chromosome 2q31 [26]. KIAA1715, alternatively called LNP (Lunapark), is reported at OMIM (OMIM 610236) and usually found in adult brain regions. Although no evidences were reported with this gene related to AMD in the literature, it may be a plausible candidate gene associated with AMD.

## Conclusions

Detecting epistatic interactions associated with complex and common diseases has become an important issue in human genetics and can improve prevention, diagnosis and treatment of those diseases. GWAS provides a huge amount of whole genomic data and therefore an unprecedented opportunity to identify causal genes or SNPs for some complex diseases. Traditional statistical methods, however, are not suitable for dealing with large datasets because of their computational complexity. Machine-learning approaches can be scaled to large datasets, but most existing machine-learning methods do not consider the complexity of genetic mechanisms and only focus on the selection of SNPs sets, which show the best classification capacity. This will introduce many false positives inevitably.

In this paper, we use a Markov Blanket-based method, DASSO-MB, to identify epistatic interactions. We compared DASSO-MB with four other methods, BEAM, Support Vector Machine, MDR and stepwise penalized logistic regression over simulated datasets. Our results show that the DASSO-MB algorithm outperforms other methods in terms of the power. It can identify a minimal set of SNPs associated with diseases, which contains less false positives. This is critical in saving the potential costs of biological experiments and being an efficient guideline for pathogenesis research.

## Methods

### Markov Blanket

Bayesian networks are probabilistic graphical models representing a joint probability distribution *J* over a set of random variables *X*_1_,*X*_2_…,*X_n_* by a directed acyclic graph (DAG) *G* and encode the Markov condition property: each node is conditionally independent of its non-descendents given its parents [27]. In this case, the joint probability distribution *J* can be represented as(1)

where *Pa*(*X_i_*) denotes the set of parents of *X_i_* in *G*.

For three random variables *X*, *Y* and *Z*, if the probability distribution of *X* conditioned on both *Y* and *Z* is equal to the probability distribution of *X* conditioned only on *Y*, i.e., *P*(*X*|*Y*,*Z*) = *P*(*X*|*Y*), *X* is conditionally independent of *Z* given *Y*. This conditional independence is represented as. Similarly, represents conditional dependence.

**Definition 1 (Faithfulness)***A Bayesian network N and a joint probability distribution J are faithful to each other if and only if every conditional independence entailed by the DAG of N and the Markov Condition is also present in J *[28].

**Figure 2:**
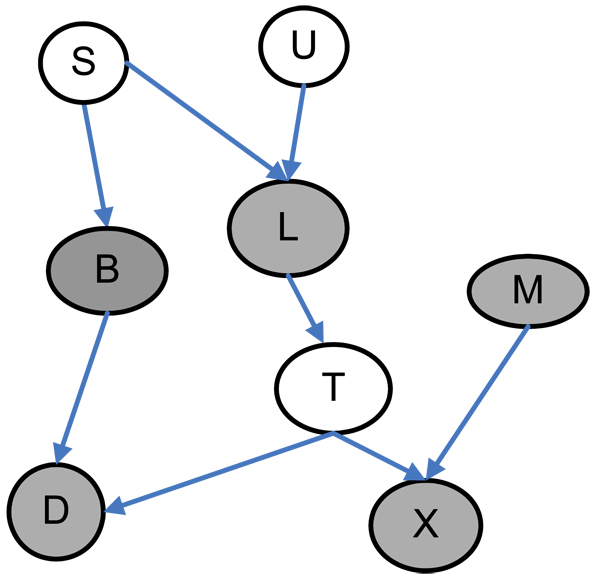
**Markov Blanket in a Bayesian Network** The gray-filled nodes are the Markov Blanket of node T.

We can define the Markov Blanket of a target variable of *T*, *MB(T)*, as a minimal set for which , for all  where *V* is the variable set in Bayesian network *N*. The Markov Blanket of a variable *T* is a minimal set of variables which can completely shield variable *T* from all other variables. All other variables are probabilistically independent of the variable *T* conditioned on the Markov Blanket of variable *T*. We show an example of the Markov Blanket in Figure 2. The *MB(T)* of the variable *T* is the set of gray-filled nodes *{B, L, M, D, X }* and variable *S* and *U* are independent of *T* conditioned on *{B, L, M, D, X }*.

**Theorem 1.***If Bayesian network N is faithful to its corresponding joint probability distribution J, then for every variable T, MB(T) is unique*.

Given the definition of Markov Blanket, the probability distribution of *T* is completely determined by the values of variables in *MB*(*T*). Therefore, the detection of Markov Blanket has been applied for optimal variable selection problem [29]. In addition, the Markov Blanket can be used for causal discovery because *MB* (*T*) is the union of direct cause variables (parents), direct effect variables (children), and direct cause variables (spouse) of direct effect variables of *T*. Thus the Markov Blanket learning method is suitable for detection of epistatic interactions in genome-wide case-control studies, e.g., to identify a minimal set of SNPs which may cause the disease for further experiments.

### *G*^2^ Test

The *G*^2^ test is commonly used to test independence and conditional independence between two variables for discrete data as an alternative to the *X*^2^ test because *G*^2^-values are additive and can be applied to more complicated statistical designs [28,30,31]. The null hypothesis for *G*^2^ test is that the two variables are independent.

Assume that we have a contingency table to record and analyze the joint distribution of two variables. The count in a particular cell in a contingency table, *x_ij_*, is the value of a random variable from *N* samples with a multinomial distribution. Let  represent the sum of elements in all cells along the *i *th row, and  denote the sum of the counts in all cells along the *j *th column. If these two variables are independent based on the null hypothesis, the expected value of the random variable *x_ij_* is:(2)

We can compute the conditional independence from appropriate marginal distributions in a similar way. For instance, to determine whether the first variable is independent of the second conditioned on the third, we can calculate the expected value of a cell *x_ijk_* as(3)

For *n* cells in a contingency table, assume that the observed numbers are denoted by *O_1_*, *O_2_*, …, *O_n_* and the corresponding expected numbers by *E_1_*, *E_2_*, …, *E_n_*, then, the *G*^2^ is given by       (4)

which has an asymptotical distribution as chi-square (*X*^2^) with appropriate degrees of freedom. The degrees of freedom (*df*) for the *G*^2^ test between two variables A and B can be calculated as:

*df* = (*Cat*(*A*) – 1)×(*Cat*(*B*)-1)          (5)

and the degrees of freedom (*df*) for the *G*^2^ test between A and B conditional on the third variable C can be calculated as:(6)

where *Cat(X)* is the number of categories of the variable *X* and *n* is the number of variables in C. Here in (5) and (6) we assume that there are no empty cells in the contingency table. If there are some empty cells in the contingency table, we should reduce the degrees of freedom from (5) or (6) by the number of empty cells.

As described next, the proposed DASSO-MB uses *G*^2^ to test the association and independence between SNPs and disease status.

### DASSO-MB

**Figure 3:**
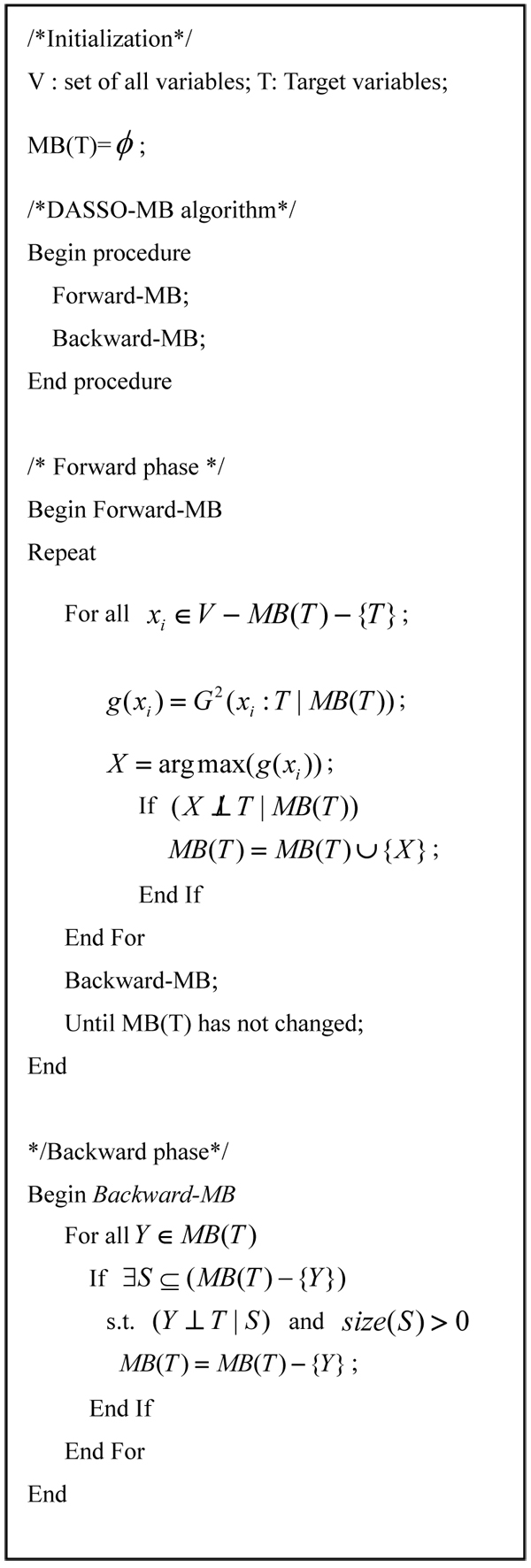
DASSO-MB algorithm

We use a Markov Blanket-based algorithm, DASSO-MB, to detect gene-gene interactions (Figure 3). Let T denote the disease status and V the set of all variables containing T and all SNPs. There are two types of phases in DASSO-MB: forward phase and backward phase. In each loop of the forward phase, if one variable shows a maximal *G*^2^ score conditioned on *MB*(*T*) and is dependent on target variable *T* , it will be admitted into *MB*(*T*). This admission operation is followed by a backward phase to remove false positives by conducting conditional independence tests. If no more variable will be added into *MB*(*T*) in the forward phase, we will enter the final backward phase to remove variables that do not belong to *MB*(*T*).

There are several methods to find the Markov Blanket for the target *T*: KS algorithm [32], GS algorithm [33], IAMB [34], MMMB [35] and HITON-MB [29]. Different Markov Blanket methods have their own advantages and disadvantages. For example, IAMB is computationally efficient, but tends to include some false positives and is not sample-efficient. Comparing to IAMB, DASSO-MB adds a backward phase after each step of selecting a variable in the forward phase to remove false positives, make the size of MB(T) as small as possible and therefore improve the sample-efficiency. In addition, it uses subset *S* of *MB* (*T*) rather than the remaining set *MB* (*T*) - {Y} while conducting the conditional independence tests in the backward phase. Here we let the size of subset *S* of *MB* (*T*) be larger than zero and exclude the empty set because of the joint effect of set of SNPs on the disease status. These two changes can make the detected results more reliable.

## List of abbreviations used

GWAS: genome-wide association studies; DASSO-MB: Detection of ASSOciations using Markov Blanket; SNP: single nucleotide polymorphisms; MDR: multifactor dimensionality reduction; stepPLR: stepwise penalized logistic regression; BEAM: Bayesian epistasis association mapping; MCMC: Markov Chain Monte Carlo; RFE: recursive feature elimination; RFA: recursive feature addition; GA: genetic algorithm; SWSFS: Sliding Window Sequential Forward feature Selection; AMD: Age-related Macular Degeneration; MAF: minor allele frequencies; LD: linkage disequilibrium; HWE: Hardy-Weinberg Equilibrium; DAG: directed acyclic graph.

## Competing interests

The authors declare that they have no competing interests.   

## Authors' contributions

BH designed and implemented the DASSO-MB method. BH and MP participated in testing the existing methods and analyzing experimental results. XWC conceived the study and designed the experiments. All authors helped in drafting the manuscript and approved the final manuscript.
